# Primary Dural Lymphoma: A Rare Case With Rapidly Growing Lesions and Successful Treatment Through Biopsy and Chemotherapy

**DOI:** 10.7759/cureus.93761

**Published:** 2025-10-03

**Authors:** Aya Endo, Katsuya Komatsu, Yukinori Akiyama, Ryohei Saito, Takeshi Mikami, Nobuhiro Mikuni

**Affiliations:** 1 Department of Neurosurgery, Sapporo Medical University, Sapporo, JPN

**Keywords:** biopsy, diffuse large b-cell lymphoma, malignant lymphoma, non-hodgkin’s lymphoma, primary dural lymphoma

## Abstract

Primary dural lymphoma (PDL) is an exceptionally rare subtype of lymphoma restricted to the central nervous system. Owing to its lack of distinctive clinical symptoms and radiographic features, it often poses a diagnostic challenge, particularly in differentiating it from meningioma. Here, we report a case of malignant lymphoma in the left parietal region that rapidly enlarged and was difficult to diagnose. Magnetic resonance imaging (MRI) revealed a lesion extending from the subcutaneous region to the extradural and intradural regions. In cases of rapidly growing disease, lymphoma should be aggressively considered and preceded by a biopsy; this will appropriate chemoradiation with minimal surgical invasion.

## Introduction

Primary malignant lymphoma of the central nervous system (PCNSL) is a non-Hodgkin’s lymphoma that occurs exclusively in the CNS, and its subtype, primary dural lymphoma (PDL), a very rare disease, accounts for approximately 1% of all cerebral lymphomas [1]. Primarily located in the convexity region, PDLs also occur in the midbrain, subcranial region, ventricles, and spinal dura mater [2, 3]. Clinically, it often presents with nonspecific symptoms such as headache or focal neurological deficits. They are difficult to diagnose on magnetic resonance imaging (MRI) alone because the lesions may appear hypointense on T1-weighted imaging [4-7] and hyperintense on T2-weighted imaging, and a dural tail sign can also be observed, as with meningiomas [4,6]. Therefore, histopathological confirmation is essential for accurate diagnosis and appropriate treatment planning. In this report, we describe a case of rapidly growing PDL with subcutaneous, extradural, and intradural extensions in which a biopsy was performed first with a good outcome.

This article was previously presented as an oral presentation at the 87th Annual Meeting of the Hokkaido Branch of the Japan Neurosurgical Society on March 26, 2022.

## Case presentation

**Figure 1:**
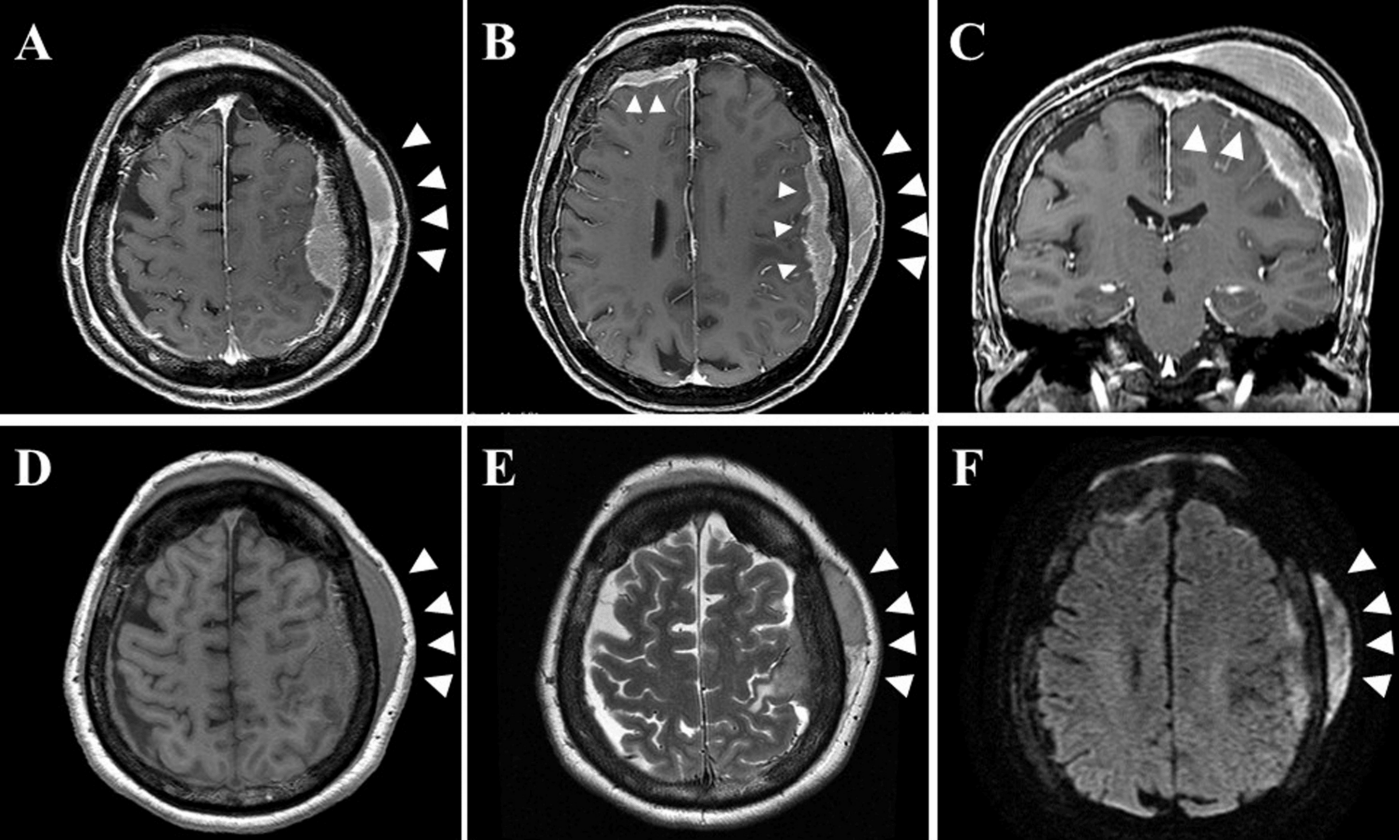
Magnetic resonance images (MRI) Axial contrast-enhanced T1-weighted image showing a mass lesion with extensive intracranial and extracranial enhancement (arrowhead) (A). Axial image showing a dural lesion extending continuously from the right frontal to left parietal region (arrowhead) (B). Coronal image showing dural tail sign (arrowhead) (C). Axial T1-weighted image showing an isointense lesion (D). Axial T2-weighted image showing the lesion as slightly hyperintense (E). Diffusion-weighted image showing the lesion as hyperintense (F).

**Figure 2:**
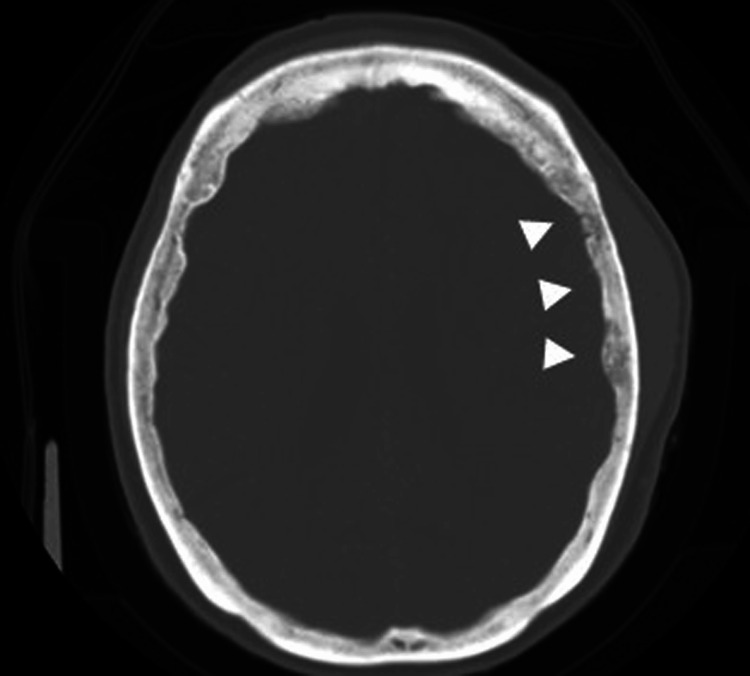
Computed tomography Computed tomography of the bone revealed no bone destruction or calcification (arrowhead).

A 69-year-old woman visited our hospital with the chief complaint of rapid swelling in the frontal and parietal regions of her head; the swelling had significantly increased in size over the past two months. Head MRI was performed; this revealed a mass lesion with extensive intracranial and extracranial involvement. A needle biopsy was performed but did not lead to a definitive diagnosis; therefore, the patient was referred to our department for further examination and treatment. Upon admission, the patient was conscious and had an elastic hard mass in the left parietal region. Mild paralysis of the right upper extremity (Manual Muscle Testing score, 4/5) and impaired dexterity of the right hand were observed. Blood tests showed a high soluble interleukin (IL)-2 receptor level of 1070 U/mL (reference range: 157-475 U/mL) and CA15-3 level of 25.1 U/mL (reference range: 0-25 U/mL), but no other abnormal findings. On imaging studies, the contrast-enhanced MRI T1-weighted image showed a large mass lesion with extensive intracranial and extracranial contrast (Figure 1A), and the dural lesion showed continuity from the right frontal to the left parietal region (Figure 1B), with a dural tail sign (Figure 1C), representing thickening of the dura adjacent to the tumor. The lesion was isointense to the brain parenchyma on T1-weighted images (Figure 1D), slightly hyperintense relative to the parenchyma on T2-weighted imaging (Figure 1E), and hyperintense on diffusion-weighted cortical imaging (Figure 1F). Despite the extensive dural infiltration, computed tomography (CT) analysis of the bone revealed no bone destruction or calcification (Figure 2).

**Figure 3:**
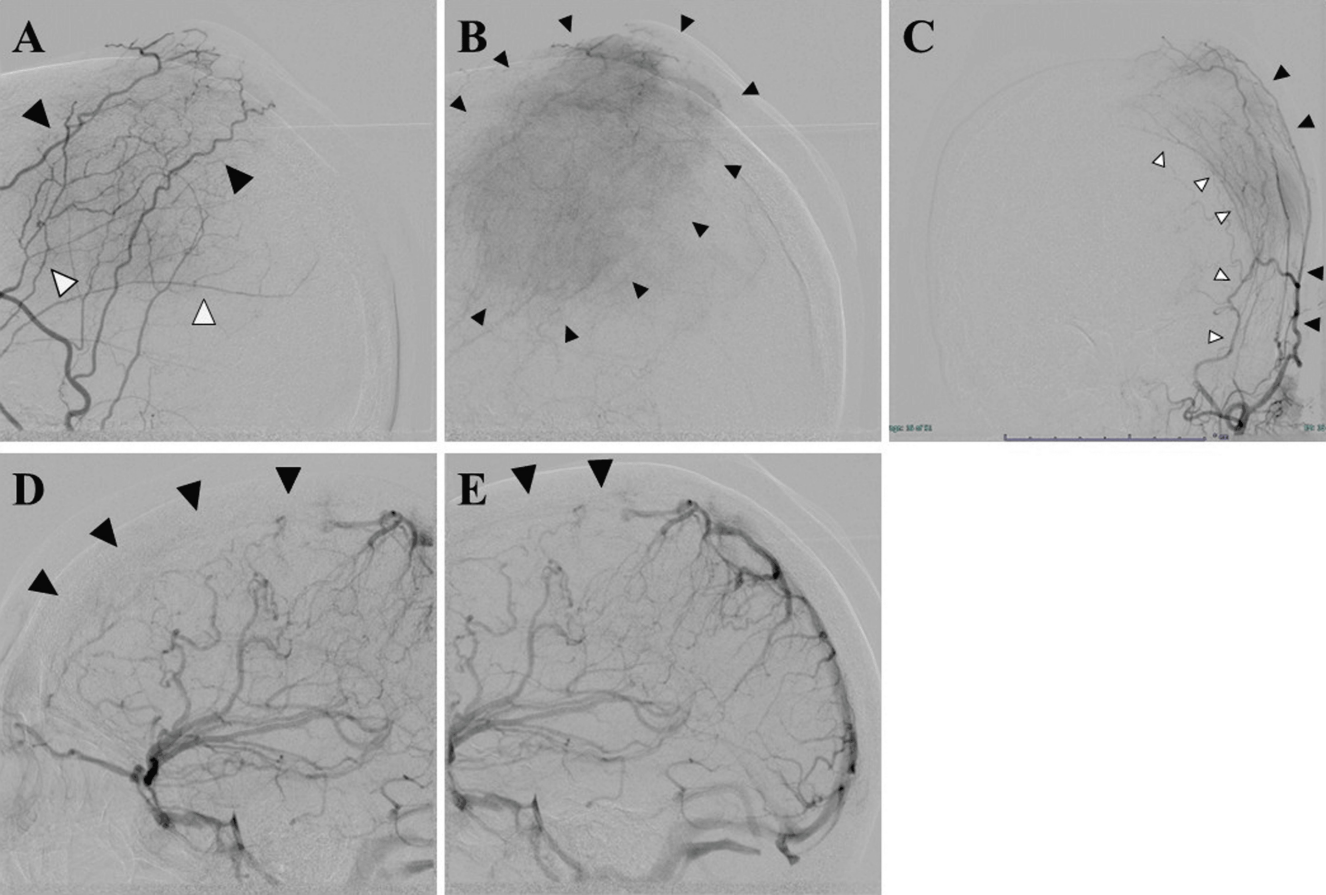
Angiography Left external carotid arteriography revealed a slightly-stained tumor shadow from the superficial temporal (black arrowhead) and middle meningeal arteries (white arrowhead) (lateral [A, B] and anterior-posterior [C] views). The venous phase on the left internal carotid arteriography showed occlusion of the superior sagittal sinus (black arrowhead, D, E).

Because the lesion was large and imaging suggested occlusion of the superior sagittal sinus, cerebral angiography was performed to evaluate venous drainage and tumor vascularity. Angiography revealed a slightly-stained tumor shadow in the superficial temporal and middle meningeal arteries on left external carotid arteriography (Figures 3A-3C), suggesting that these vessels contributed to the tumor’s blood supply. Furthermore, the venous phase of the internal carotid arteriography revealed occlusion of the superior sagittal sinus (Figures 3D, 3E).

**Figure 4:**
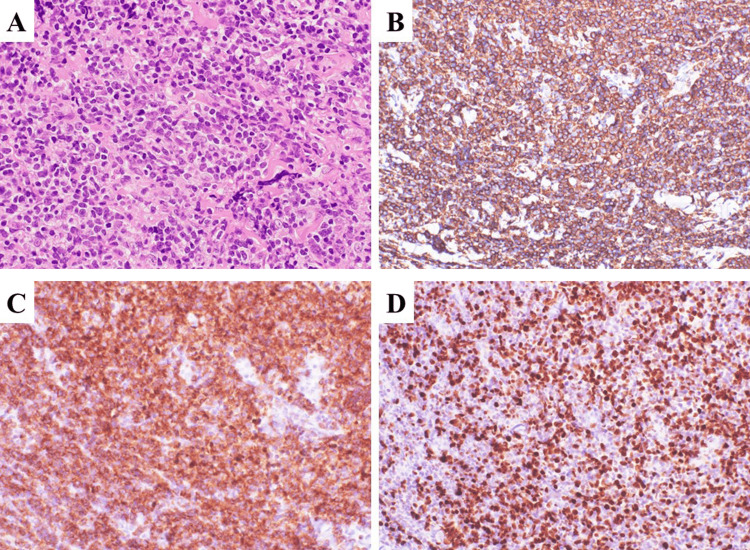
Immunopathological findings Diffuse growth of chromatin-rich tumor cells with a high nucleus-to-cytoplasm (N/C) ratio was noted (hematoxylin and eosin staining, ×40; A). The tumor cells were positive for CD20 (B; ×20) and CD79α (C; ×20). The proportion of Ki-67-positive cells was more than 80% (D; ×20). The final pathological diagnosis was diffuse large B cell lymphoma.

Based on these radiological findings, meningioma was considered among the differential diagnoses. For a conclusive diagnosis, we performed a biopsy under local anesthesia 1 week after admission, focusing on the primary lesion located beneath the skin in the left parietal region. Upon making an incision in the skin, we discovered a tumorous lesion directly below the galea. We collected a sample from the lesion for pathological examination. As a pathological finding, Hematoxylin-eosin staining revealed diffuse　proliferation of chromatin-rich, high-nucleus-to-cytoplasm (N/C) tumor cells that resembled lymphocytes. The tumor cells were conspicuous because of their irregular nuclear shape and small and large nuclei with large endothelial vessels (Figure 4A). Immunohistochemistry revealed that the tumor cells were positive for CD20 (Figure 4B) and CD79α (Figure 4C) and >80% of the cells were positive for Ki-67 (Figure 4D).

**Figure 5:**
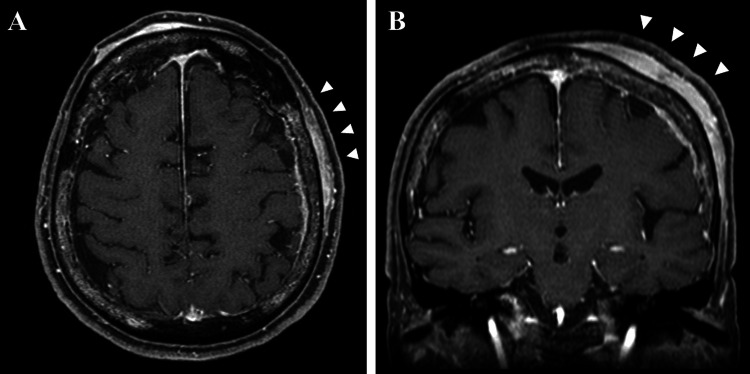
Contrast-enhanced MRI after the first course of multiagent chemotherapy Marked tumor shrinkage was observed (arrowhead, axial (A), and coronal view (B)).

The final pathological diagnosis was diffuse large B-cell lymphoma (DLBCL). The patient was transferred to the Department of Hematology at our hospital on day 20 for treatment. After the first course of multiagent chemotherapy consisting of rituximab, high-dose methotrexate, procarbazine, and vincristine, contrast-enhanced MRI was performed, and marked tumor shrinkage was noted, with near-complete resolution of the intracranial component and a reduction in thickness of the extracranial lesion (Figures 5A, 5B). The patient was transferred to another hospital for the continuation of chemotherapy. Two years have passed since the start of treatment and the tumor has not recurred.

## Discussion

A type of non-Hodgkin’s lymphoma that occurs exclusively in the parts of the CNS, including the brain parenchyma, meninges, spinal cord, cranial nerves, and orbital structures, PCNSL accounts for 1-6% of all intracranial tumors [8,9]. A very rare subtype of PCNSL, PDL, accounts for less than 1% of all cerebral lymphomas. While PCNSL mostly represents a high-grade DLBCL, PDL is a low-grade marginal zone B-cell lymphoma that is assumed to have a better prognosis than PCNSL within the brain parenchyma [10-14]. The main site of predilection is the convexity region; however, PDL can also occur in the falx, skull base, suprasellar region, and spinal dura mater [11,15]. PDL presents with a variety of local symptoms such as headache, seizures, dizziness, weakness, numbness, and ataxia, all of which are nonspecific and depend on the site of origin [4,6,10,11,15]. In some instances, lesions are observed both intracranially and subcutaneously. In such cases, they may present as masses growing rapidly on the head over several months [13,16,17].

Imaging features of PDL are also nonspecific. Typically, lesions appear iso- to hypointense on T1-weighted images relative to the brain parenchyma, iso- to hyperintense on T2-weighted images relative to the brain parenchyma, and often show heterogeneous enhancement on gadolinium contrast [4-7,13,18]. Depending on the size and location of the tumor, fluid-attenuated inversion recovery (FLAIR) images often show mild brain edema around the tumor [7,11]. The lesion may appear hyperintense relative to the normal white matter on diffusion-weighted imaging (DWI), indicating a high cellular density [4,11,18]. Patients with and without bone destruction have been reported [4,10,13,16,18]. PDL is often difficult to diagnose on imaging due to its nonspecific clinical and radiological features, making differentiation from meningioma particularly important [12-15,18]. In addition to meningiomas, there have been reports of cases in which it was difficult to diagnose PDL from dural metastases of the tumor, solitary fibrous tumors, gliosarcomas, leiomyosarcomas, or epidural hematomas [18,19]. A standard treatment for PDL has not yet been established because the disease is very rare. Current treatment options include surgical resection, chemotherapy, and radiation therapy [4,6,10,11,13-15], among which the response to chemotherapy is considered favorable and is the mainstay [12,13,15,18]. Notably, most cases of PDL are diagnosed after invasive craniotomy; however, patients have been reported to respond well to chemotherapy [12,13,15]. In the present case, owing to the presence of intracranial involvement and the possibility of CNS invasion, treatment was initiated with a regimen that included methotrexate, resulting in marked tumor shrinkage and a recurrence-free course for two years.

In previous reports on PDL, most cases involved extensive craniotomies, and we could not find any reports of an initial biopsy [4,5,12,13,15,16]. In cases of suspected invasive meningioma, extensive craniotomy was performed because contrast-enhanced MRI showed a contrast lesion with a dural tail, and angiography showed nutrient vessels originating from the external carotid artery [16]. In another case of a suspected epidural hematoma, intraoperative pathology was performed; however, a craniotomy was performed to reduce the mass effect [20]. Notably, in the present case, the only symptoms were a skin mass, mild paralysis of the right upper extremity, and impaired dexterity of the right hand, while imaging findings alone were inconclusive, and meningioma was considered in the differential based on a series of examinations. Because the lesion was extensive and showed rapid growth within a short period, an initial biopsy enabled the diagnosis of PDL. Tissue diagnosis of tumors is the gold standard, and the possibility of PDL should be considered in cases of suspected extensive meningiomas.

## Conclusions

Primary dural lymphoma is a rare disease with nonspecific symptoms that can be difficult to distinguish from meningiomas on imaging studies. Histopathological confirmation is essential for definitive diagnosis. In cases of rapidly growing lesions and suspected meningioma or malignant lymphoma, a biopsy should be performed first. PDL has been reported to respond well to chemotherapy, and early biopsy with minimally invasive intervention is particularly important for the timely initiation of treatment.
